# RGD Peptide-Conjugated Selenium Nanocomposite Inhibits Human Glioma Growth by Triggering Mitochondrial Dysfunction and ROS-Dependent MAPKs Activation

**DOI:** 10.3389/fbioe.2021.781608

**Published:** 2021-12-23

**Authors:** Wenjian Liu, Jing Su, Qiang Shi, Jinlei Wang, Xiao Chen, Shizhong Zhang, Mengkao Li, Jie Cui, Cundong Fan, Beibei Sun, Guojun Wang

**Affiliations:** ^1^ Department of Oncology, Second Affiliated Hospital of Shandong First Medical University, Shandong Academy of Medical Sciences, Taian, China; ^2^ Department of Geriatrics, Taian City Central Hospital, Taian, China; ^3^ Department of Internal Medicine, Taian Traffic Hospital, Taian, China; ^4^ Department of Neurosurgery, Taian City Central Hospital, Taian, China

**Keywords:** glioma, apoptosis, mitochondrial dysfunction, reactive oxygen species, MAPKs

## Abstract

Chemotherapy is still one of the most common ways to treat human glioblastoma in clinic. However, severe side effects limited its clinic application. Design of cancer-targeted drugs with high efficiency and low side effect is urgently needed. Herein, silver nanoparticles (Ag NPs) and nano-selenium (Se NPs) conjugated with RGD peptides (Ag@Se@RGD NPs) to target integrin high-expressed glioma were designed. The results found that Ag@Se@RGD NPs displayed stable particle size and morphology in physiological condition, and induced significant integrin-targeted intracellular uptake. Ag@Se@RGD NPs *in vitro* dose-dependently inhibited U251 human glioma cells growth by induction of cells apoptosis through triggering the loss of mitochondrial membrane potential, overproduction of reactive oxygen species (ROS), and MAPKs activation. However, ROS inhibition dramatically attenuated Ag@Se@RGD NPs-induced MAPKs activation, indicating the significant role of ROS as an early apoptotic event. Importantly, Ag@Se@RGD NPs administration *in vivov* effectively inhibited U251 tumor xenografts growth by induction of apoptosis through regulation MAPKs activation. Taken together, our findings validated the rational design that Ag-Se NPs conjugated with RGD peptides was a promising strategy to combat human glioma by induction of apoptosis through triggering mitochondrial dysfunction and ROS-dependent MAPKs activation.

## Introduction

Glioma is still considered as the most common primary malignant brain tumors, accounting for about 80% of malignant brain tumors (Gittleman et al., 2020). Chemotherapy as one of the most commonly used cancer treatments is severely affected by drug dosage and drug toxicity (Deng et al., 2015; Sun et al., 2021). The prognosis of gliomas is often poor, and chemotherapy resistance remains the challenge in therapy of human glioma (da Silva., et al., 2020; Ding et al., 2020; Guo et al., 2021). A safe dose of chemotherapeutic drugs may not cure cancer patients, while high-dose drugs have a more significant therapeutic effect but higher drug toxicity and side effects (Song et al., 2016; Sun et al., 2020). For example, cisplatin is used as a broad-spectrum anti-tumor drug by inhibiting cell DNA replication and damaging its cell membrane structure. However, cisplatin may damage surrounding healthy tissues and cause severe nephrotoxicity and bone marrow toxicity (Cemazar et al., 1999; Plummer et al., 2011; Volarevic et al., 2019). Therefore, the tumors may not be completely eradicated, which may lead to tumors recurrence and metastasis. It is urgently needed to develop a targeted, low effective dose and low toxicity chemotherapeutic drug to inhibit tumor cell proliferation (Fang et al., 2011; Nakamura et al., 2015; Cai et al., 2021).

Nanoparticles recently have attracted much attention in the field of cancer treatment due to their special physical and chemical properties (Wang et al., 2011; Li et al., 2018). Compared with traditional anti-cancer drugs, metal nanoparticles can be used as new therapeutic drugs or drug carriers in combination with candidate drugs, and targeted therapy showed less side effects (Zhao et al., 2016; Huang and Huang, 2018; Zhang et al., 2018). Wang et al. found that fructose-modified nano-silver can enter and accumulate in a variety of cancer cells to induce apoptosis, but show less toxicity to most normal cells (Wang Z. X. et al., 2019). Increasing studies have found that nano-silver exerts broad-spectrum anti-tumor activity through a variety of mechanisms. Nano-silver destroys the ultrastructure of cancer cells, induces ROS production and DNA damage and leads to cell apoptosis. Nano-silver can also reduce tumor metastasis by inhibiting tumor cell migration and angiogenesis (Liu et al., 2018; Zhu et al., 2019; Alphandery 2020; Yang et al., 2021).

Accumulated researches indicated that selenium was an necessary element and nano-selenium was an effective anti-tumor nano-drug and drug carrier (Alvarez et al., 2021). The team also discovered that tumor-targeted proteins are used to modify nano-selenium, which improved the stability and tumor targeting of nano-selenium (Deng et al., 2015; Chang et al., 2017). Therefore, the modified nano-selenium can achieve precise drug delivery and inhibit tumor growth and migration. RGD peptides can bind to integrins that are specifically expressed in tumor cells or new blood vessels, such as α_v_β_3,_ but the content in blood vessels of normal tissues is very low (Danhier et al., 2012; Zhong et al., 2014; Chen et al., 2015). Therefore, such receptors can be used as targets for tumor-targeted therapy, and exogenous RGD peptides can bind integrin receptors, inhibit tumor migration and tumor new blood vessel formation, and can also target the delivery of anti-tumor drugs (Choi et al., 2013; Xu et al., 2017; Wang P. et al., 2019).

Herein, silver nanoparticles (Ag NPs) and nano-selenium (Se NPs) conjugated with RGD peptides (Ag@Se@RGD NPs) were synthesized, and our findings validated the rational designs that Ag-Se NPs conjugated with RGD peptides was a promising strategy to combat human glioma by induction of apoptosis through triggering mitochondrial dysfunction and ROS-dependent MAPKs activation.

## Experimental Section

### Materials

Silver nitrate (AgNO_3_), polyvinylpyrrolidone (PVP), ethylene glycol, glycerol, sodium selenite (Na_2_SeO_3_), chitosan (Cs, molecular weight 150 kDa; deacetylation degree 85%), sodium chloride, l-ascorbic acid (Vc), 1-ethyl-3-[3-(dimethylamino)-propyl] carbodiimide hydrochloride (EDC), N-hydroxysuccinimide (NHS) were purchased from Sigma-Aldrich. RGD (H-Gly-Arg-Gly-Asp-Asn-Pro-OH) were purchased from Sangon Biotech (Shanghai) Co., Ltd. Fluorescein isothiocyanate (FITC), 2,7-Dichlorodihydrofluorescein diacetate (DCFH-DA), live/dead cell viability kit, BCA protein detection kit, mitochondrial membrane potential detection kit (JC-1), apoptosis detection kit (annexin V-FITC and propidium iodide) were purchased from ThermoFisher Scientific (China) Co., Ltd. All antibodies were obtained from Cell Signal Technology (United States).

### Synthesis of Ag NPs

The silver nanosphere colloidal solution was prepared as followed. Briefly, silver nitrate (100 mg) and PVP (1.5 g) with 15 ml ethylene glycol were mixed, vigorously stirred and heated in an oil bath at 120 °C. After reaction 4 h, the reaction solution gradually turns yellow-green, and the reaction solution was rapidly reduced to room temperature to obtain a nano silver colloidal solution.

### Preparation of Target Substance Cs-RGD

RGD (10 mg/ml) was incubated with NHS and EDC for 2 h. Cs (1 mg/ml) was dissolved in 1% acetic acid solution and was stirred for 30 min. Then, Cs solution was added to the RGD solution, reacted overnight, and dialysis was used to remove unreacted substances to obtain Cs-RGD. Un-reacted NHS, EDC and RGD with a molecular weight below 8–14 kDa were all removed.

### Synthesis of Ag@Se@RGD NPs

Ag NPs colloidal solution (0.5 ml) was added to Na_2_SeO_3_ solution (10 ml, 1 mg/ml), and Vc (1.2 ml, 35 mg/ml) was slowly added with constant stirring. After 15 min reaction, Cs solution (1 ml, 1 mg/ml) and Cs-RGD solution (1 ml, 2 mg/ml) were added and reacted for 12 h. Then, dialysis was performed to remove un-reacted substances. The Ag@Se@RGD NPs were removed from the reaction solution by centrifugation (10,000 g/min, 10 min). Supernatant was collected, and BCA detection kit was used to detect the residual peptides, and the conjugated content of peptide (μg/mg NPs) was calculated. The preparation of Coumarin-6-labeled Ag@Se@RGD NPs was similar to the above preparation process.

### Characterization

The morphology of the nanoparticles was observed by high-definition transmission electron microscope (TEM) and scanning electron microscope (SEM). Element analysis was determined by energy dispersive X-ray spectroscopy (EDS). The dynamic light scattering (DLS) and zeta potentials measurements were used for characterization of NPs optical properties and sizes on a Brookhaven Zeta PALS instrument. The ultraviolet-visible absorption spectra (UV-vis) was obtained on a UV2600 spectrophotometer. Fourier transform infrared spectroscopy (FT-IR) was performed on a FT-IR spectrometer (Nicoletteis50, Thermo Fisher Scientific United States) in the wavelength range between 4,000 cm^−1^ and 500 cm^−1^.

### Stability Analysis

Ag@Se@RGD NPs suspension (200 μl) was dispersed in 2.8 ml deionized water or 10% FBS in DMEM medium for 72 h, the dispersion stability and dimensional stability of Ag@Se@RGD NPs in the biological environment were evaluated by examining the changes in absorbance and particle size, respectively (Wang et al., 2021).

### Cell Culture and Cell Viability Assay

U251 human glioma cells were obtained from American Type Culture Collection (ATCC, United States). Cells were cultured with DMEM high glucose medium supplemented with 10% FBS, 100 units/mL penicillin and 100 units/ml streptomycin. The cell were cultured at 37°C, 5% carbon dioxide and 95% relative humidity in an incubator. The toxicity of 0–60 μg/ml Ag@Se NPs and Ag@Se@RGD NPs to Glioma cells was examined by standard MTT analysis (Sun et al., 2020).

### Intracellular Uptake of Ag@Se NPs and Ag@Se@RGD NPs

Intracellular uptake of NPs by Glioma cells was quantitatively evaluated. Briefly, U251 cells were cultured in 96-well plates at a density of 5 × 10^3^ cells/well for 24 h, and coumarin-6-labeled Ag@Se NPs and Ag@Se@RGD NPs were added and incubated for 2 h. Confocal laser microscope (Olympus, IX-71) was employed for cell imaging observation and multifunctional micro-plate reader (Tecan Infinite, 200Pro) was used to measure the fluorescence intensity of coumarin-6 (excitation wavelength = 466 nm, emission wavelength = 504 nm), respectively.

### Transmission Electron Microscope Observation of Cell Morphology

U251 cells were cultured in 6-well plates at a density of 1 × 10^6^ cells/well for 24 h. Cells were treated with 20 μg/ml Ag@Se NPs or Ag@Se@RGD NPs for 2 h. U251 cells after treatment were fixed with 2.5% glutaraldehyde overnight, and fixed with 1% osmic acid for 1 h. Then, samples were dehydrated with gradient alcohol, embedded in resin, sectioned, and stained by 1% lead citrate solution and 1% uranyl acetate solution for 10 min. Finally, the cells were fixed on a 200-mesh copper net and observed by TEM.

### Real-Time Cellular Analysis

The real-time cell electronic sensor system (RT-CES; ACEA Bioscience Company) was used to monitor cell proliferation within 72 h and recorded every 20 min (Zhang et al., 2019). Briefly, U251 cells were cultured in an e-plate at a density of 5 × 10^3^ cells per well for 24 h. Then, Ag@Se NPs or Ag@Se@RGD NPs with a final concentration at 20 μg/ml were added to the wells, and the detection was continued for 48 h.

### Live/Dead Cell Stain Detection

Briefly, U251 cells were cultured in a 6-well plate at a density of 1 × 10^6^ cells/well for 24 h. After that, Ag@Se@RGD NPs of 5, 10 or 20 μg/ml were used to treat the cells for 24 h. The cells were stained for 30 min according to the operating steps of the live/dead cell viability kit instructions, and the cell death and living status was recorded by a fluorescence microscope.

### Cellular ROS Level Detection

U251 cells were cultured in a 6-well plate at a density of 1 × 10^6^ cells per well for 24 h. Afterwards, cells were treated with 5, 10 or 20 μg/ml Ag@Se@RGD NPs for 0–2 h. Then, 10 μM DCFH-DA was used to stain the cells for 20 min. The ROS level was detected by a fluorescence microscope and a multi-function microplate reader.

### Flow Cytometry Analysis

U251 cells were cultured in 6-well plates at a density of 1 × 10^6^ cells per well for 24 h. Then, U251 cells were treated with 5, 10, or 20 μg/ml Ag@Se@RGD NPs for 24 h, and the operation was performed according to the instructions of the apoptosis detection kit and the mitochondrial membrane potential detection kit. Briefly, treated cells were stained with annexin V-FITC and propidium iodide for apoptosis detection, and treated cells were stained with JC-1 for mitochondrial membrane potential detection. Then flow cytometer (Cytoflex, Beckman) was employed to quantitatively analyze the stained cells.

### Permeability Detection of BBB Model *in vitro*

Blood brain barrier (BBB) model *in vitro* was established. Briefly, HUVECs human umbilical vein endothelial cell (1 × 10^5^ cell/well) were seeded onto the up-layer of transwell, and cultured for 24 h with 1% FBS. U251 cells (1 × 10^5^ cell/well) were seeded onto the down-layer of transwell, and cultured for 24 h with 10% FBS. HUVECs were treated with Ag@Se NPs or Ag@Se@RGD NPs (20 μg/ml) for 1 h, and then continued to be cultured under normal culture conditions. The transendothelial cell electrical resistance (TEER) value of the cell monolayer was measured within 0–24 h after adding the nanoparticles. The time-dependent permeability of the nanoparticle from up-layer to down-layer was quantified by measuring the relative light absorption of each sample.

### *In Vivo* Studies

30 nude mice (20170015) were adaptively fed for 1 weeks, and injected 10^7^ U251 cells by subcutaneous injection. After 3-weeks growth, mice (10 mice/group) were given 5 and 10 mg/kg Ag@Se@RGD NPs by caudal vein injection every other day for 2 weeks. Then, tumors were harvested, and tumors were measured and weighted. Tumors tissue were examined by immunostaining and western blotting for mechanism study *in vivo*. All animal experiments were carried out according to the protocols approved by the Guide for the Care and Use of Laboratory Animals published by Taishan Medical University (SYXK20170023).

### Western Blotting

The total cellular proteins (1 × 10^5^ cells/mL, 10 ml) treated with or without Ag@Se@RGD NPs for 0–24 h were extracted by cell lysis buffer. The protein concentration was determined by the BCA protein assay method, and 40 μg/lane protein was loaded and separated by electrophoresis. Western blotting was used to determine the effect of Ag@Se@RGD NPs on the expression level of related proteins, including p-JNK (CST, #9255), total-JNK (CST, #9252), p-ERK (CST, #3510), total-ERK (CST, #4695), p-p38 (CST, #4511), total-p38 (CST, #9212), active-caspase-3 (CST, #9661) and β-actin (CST, #4970). The target protein was detected with chemiluminescence reagents under the Bio-Rad imaging system.

### Acute Toxicity Study

In order to determine the side effects of Ag@Se@RGD NPs treatment on vital organs, 0–80 mg/kg of Ag@Se@RGD NPs was continuously administered through the tail vein, and the survival rate and body weight changes of the mice were recorded every day. After 21 days, blood samples of each group of mice were collected for analysis, and important organs such as heart, liver, spleen, lung and kidney were obtained for hematoxylin-eosin (H and E) staining.

### Statistics Analysis

All the experiments were carried out in triplicate and the data expressed as mean ± standard deviation. Statistical analysis was performed using SPSS 13.0 (SPSS, Inc.). Statistical significance was analyzed by one-way ANOVA followed by a Dunnett’s or Tukey’s post-hoc test. Signifificant differences between the treatment and control groups are indicated at *p < 0.05, **p < 0.01.

## Results and Discussion

### Synthesis and Characterization of Ag@Se@RGD NPs

**FIGURE 1:**
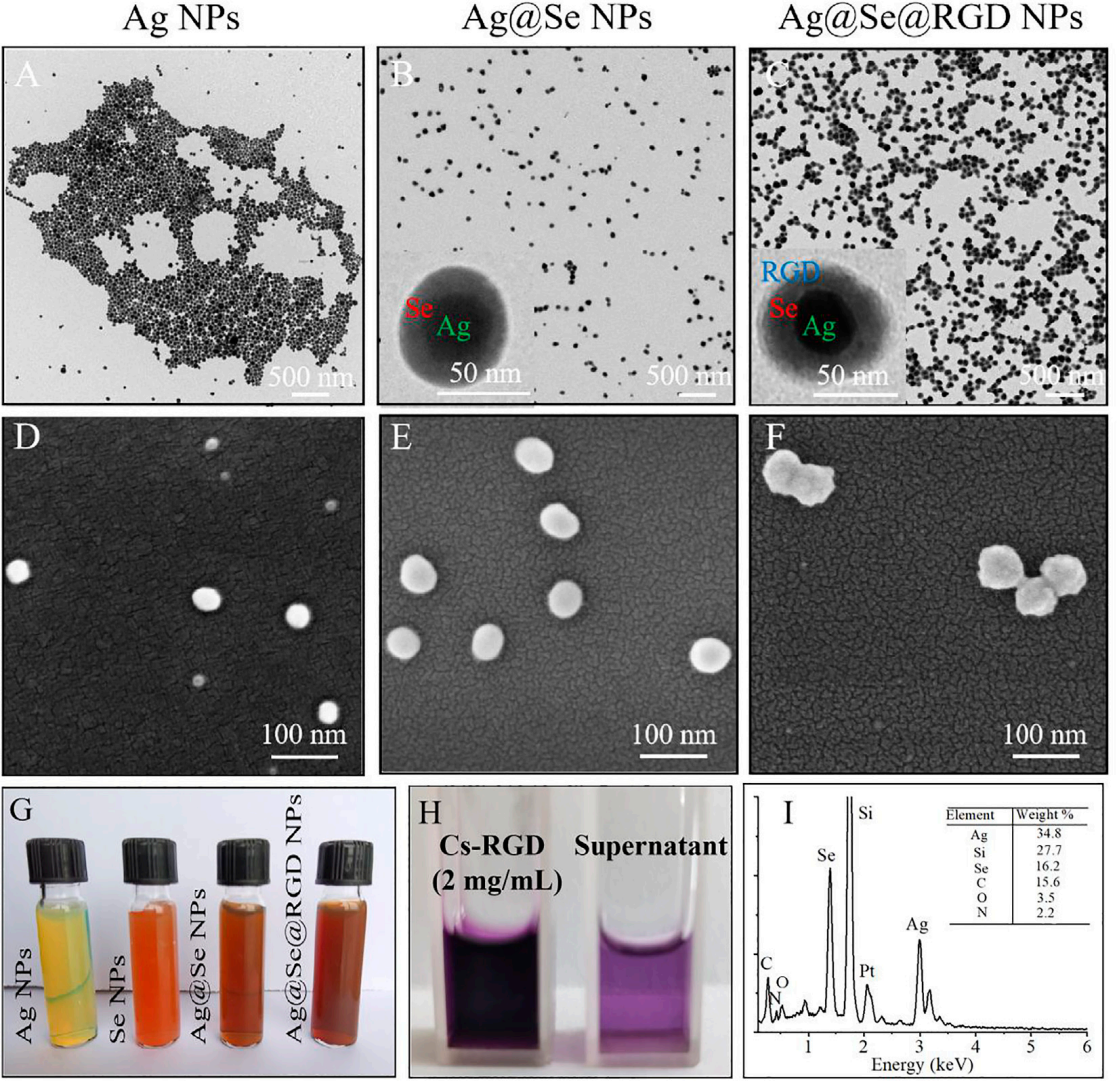
Synthesis and morphology of Ag@Se@RGD NPs: TEM images of Ag NPs **(A)**, Ag@Se NPs **(B)** and Ag@Se@RGD NPs **(C)**. SEM images of Ag NPs **(D)**, Ag@Se NPs **(E)** and Ag@Se@RGD NPs **(F)**. **(G)** Color change images of Ag NPs, Se NPs, Ag@Se NPs and Ag@Se@RGD NPs. **(H)** The difference between the added amount and the residual polypeptide in the supernatant was calculated to calculate the RGD content in NPs. **(I)** EDS spectrum of Ag@Se@RGD NPs.

**FIGURE 2:**
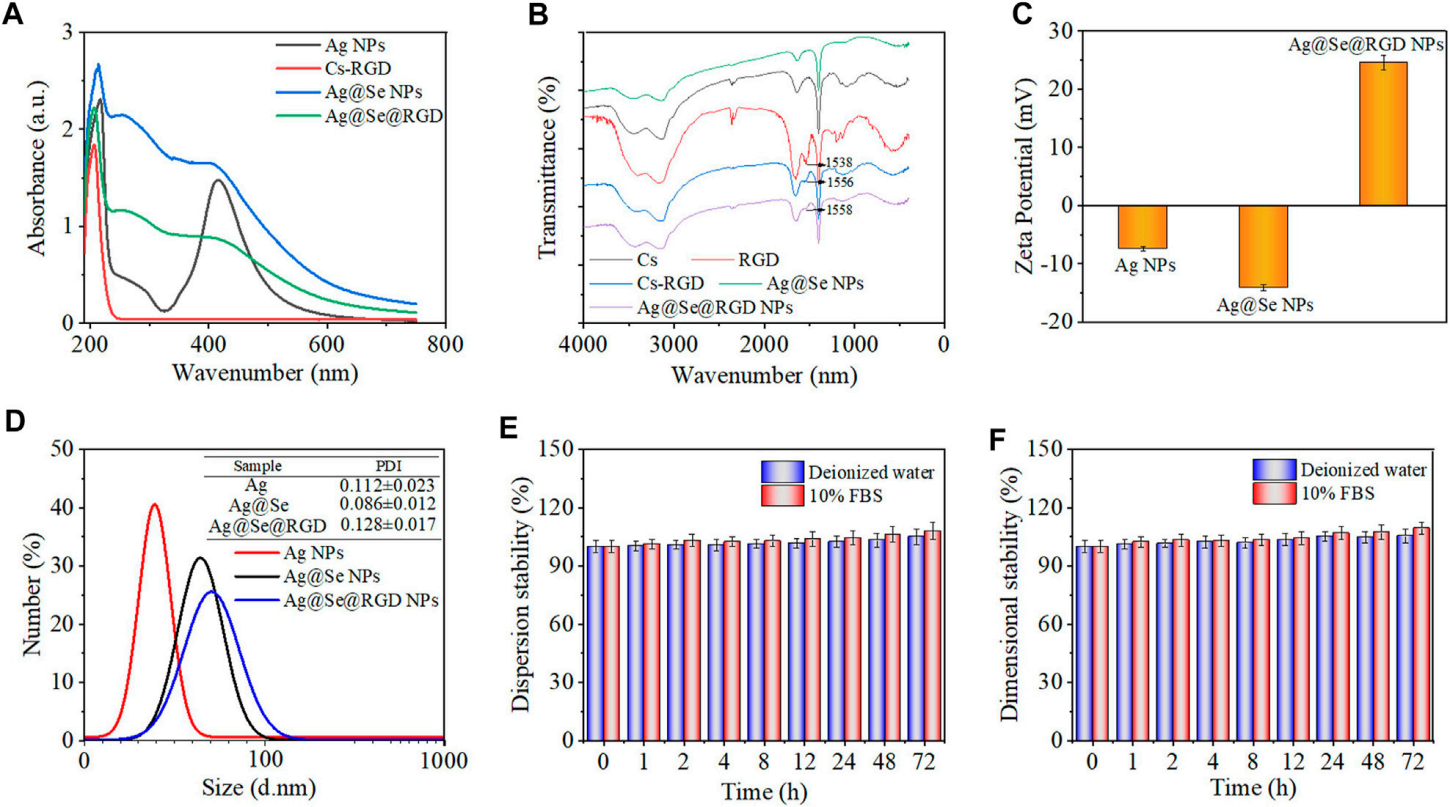
Chemical characterization of Ag@Se@RGD NPs: **(A)** UV-vis absorption spectra of Ag NPs, Cs-RGD, Ag@Se NPs and Ag@Se@RGD NPs. **(B)** FT-IR spectra of Ag NPs, RGD, Cs-RGD, Ag@Se NPs and Ag@Se@RGD NPs. **(C)** Zeta potential of different products during synthesis. **(D)** The particle size distribution of Ag NPs, Ag@Se NPs and Ag@Se@RGD NPs. The dispersion stability **(E)** and dimensional stability **(F)** of Ag@Se@RGD NPs in deionized water and 10% FBS.

The characterization results of Ag@Se@RGD NPs are shown in Figures 1A–H. PVP-assisted solvothermal synthesis was used for the synthesis of Ag NPs, and then the Ag@Se NPs with uniform size and good dispersion were obtained by reducing sodium selenite on the surface of Ag NPs. Ag NPs were spherical structures with an average diameter of 38 nm (Figures 1A,D; Figure 2D). After the formation and conjugation of the Se shell, Au@Se NPs showed a spherical shape with a uniform size about 65 nm and the Se shell was about 10 nm (Figures 1B,E; Figure 2D). Ag@Se@RGD NPs showed an obvious three-layer structure, and the average diameter increased to 72 nm (Figures 1C,F; Figure 2D). The formation and modification of the Se shell resulted in the color of the yellow-green Ag NPs colloidal solution changing to yellow-brown, and the modification of Cs-RGD did not cause a significant change in color (Figure 1G). In addition, the coupling amount of RGD in NPs was 18.32 μg/mg (Figure 1H). The elemental composition of Ag@Se@RGD NPs was further analyzed by EDS (Figure 1I). The results showed that there was a strong signal from Ag (34.80%) and Se (16.2%) in Ag@Se@RGD NPs. The presence of element N (2.2%) indicated the presence of RGD in NPs. The detected Si (27.7%) signal comes from the silicon wafer substrate. The results showed that the composite nano-system Ag@Se@RGD NPs was successfully assembled.

The typical longitudinal surface plasmon resonance band of Ag NPs was observed at 420 nm (Figure 2A), and the modification of the Se shell caused the characteristic peak at 420 nm to weaken. RGD and Cs were coupled through an acylation reaction. RGD-modified chitosan has the properties of a positively charged polyelectrolyte in an acidic medium, and can be stably combined with negatively charged Se NPs. After the modificatin of Cs-RGD, due to the increase of the refractive index of the surrounding medium, the maximum absorption wavelength has a slight red shift. The FT-IR spectral analysis results in Figure 2B showed that the characteristic peaks of RGD in Ag@Se@RGD NPs and the presence of Cs-RGD further confirmed their successful conjugation with the Se shell surface. The zeta potential analysis in Figure 2C showed that Ag NPs and Ag@Se NPs were negatively charged (−7.42 and −14.09 mV, respectively), which promoted the positively charged Cs-RGD shell to wrap, and the zeta potential changes to +24.58 mV. The dispersion stability (Figure 2E) and dimensional stability (Figure 2F) of Ag@Se@RGD NPs in the biological environment were evaluated by examining the changes in absorbance and particle size, respectively. Ag@Se@RGD NPs were dispersed in deionized water or 10% FBS and serum for 72 h, and the changes of size and sedimentation were both less than 5%, indicating the good stability in biological environment.

### Cell Uptake of Ag@Se@RGD NPs

**FIGURE 3:**
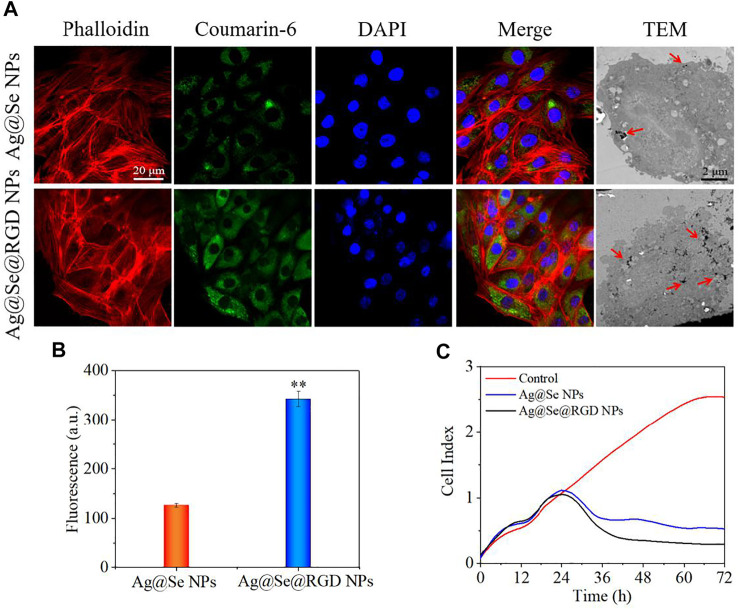
Location of Ag@Se@RGD NPs in cells and U251 cells growth inhibition: **(A)** Location of coumarin 6-labeled Ag@Se NPs and Ag@Se@RGD NPs in Glioma cells. **(B)** Quantitative analysis of fluorescence intensity. **(C)** Real-time cell analysis of the effects of Ag@Se NPs or Ag@Se@RGD NPs on the proliferation of Glioma cells.

To monitor the transport of NPs in the cell, phalloidin was used to label the cytoskeleton, and DAPI was used to label the nucleus. As shown in Figure 3A, Ag@Se@RGD NPs with green fluorescence penetrated the cell membrane and filled the entire cytoplasm within 2 h. Similar results were observed on the TEM image of the cell, which showed that a large number of nanoparticles entered the cytoplasm. On the other hand, the uptake rate of Ag@Se NPs by cells is significantly reduced, which is manifested by a significant reduction in fluorescence intensity (Figure 3B). Therefore, it was inferred that the modification of RGD increased the uptake of NPs in U251 cells.

### Ag@Se@RGD NPs Induced Apoptosis of U251 Cells

**FIGURE 4:**
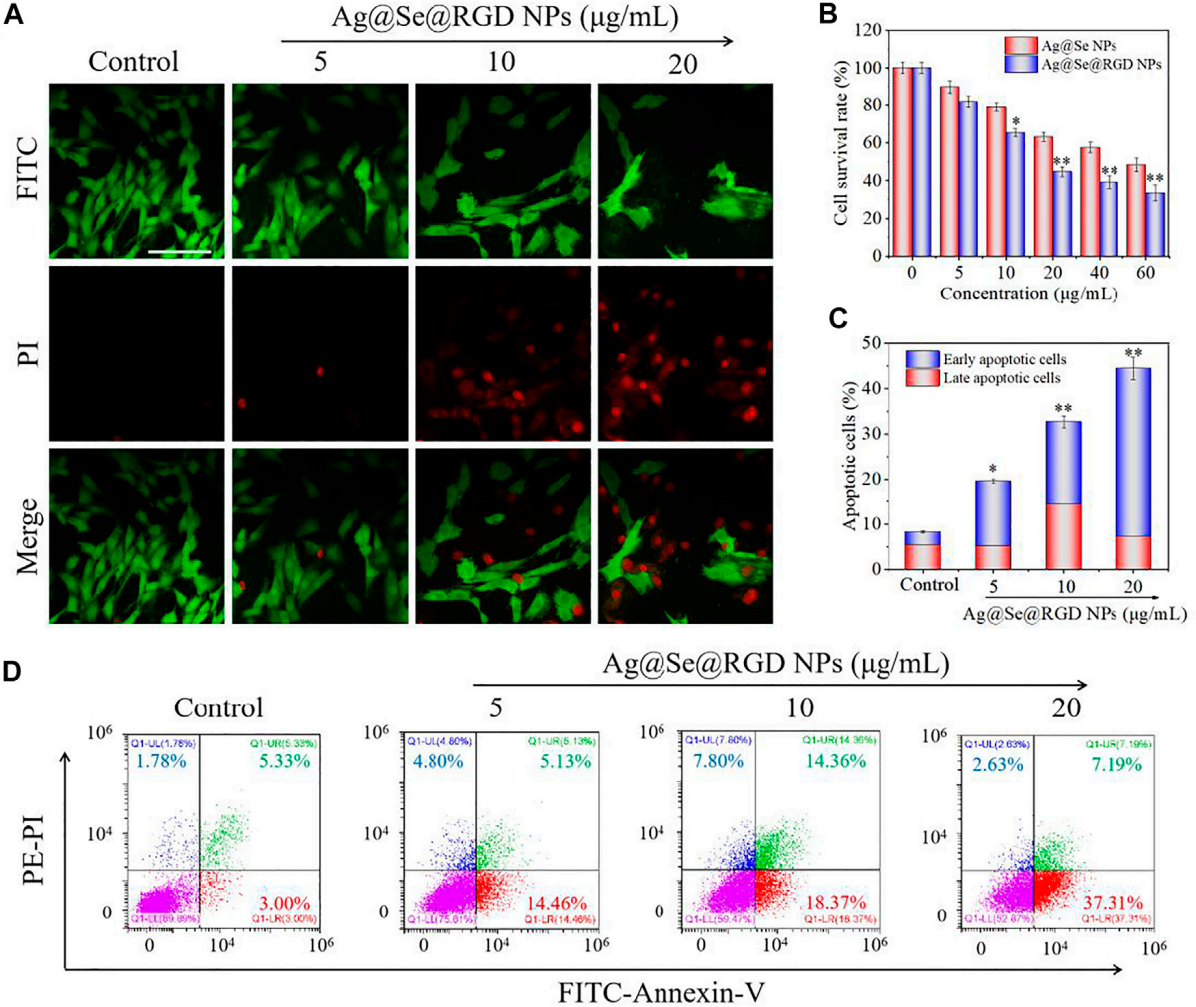
Ag@Se@RGD NPs promoted the apoptosis of U251 cells: **(A)** U251 cells treated with 5–20 μg/ml Ag@Se@RGD NPs were stained with a live/dead kit and imaged with a fluorescence microscope. Scale bar: 50 μm. **(B)** MTT was used to test the toxicity of Ag@Se@RGD NPs to U251 cells. Quantitative analysis **(C)** and flow cytometry analysis **(D)** of the early and late apoptosis of U251 cells induced by Ag@Se@RGD NPs.

Real-time cell analysis, MTT detection and live/dead cell staining were used to explore the inhibitory activity of Ag@Se@RGD NPs on U251 cells. Real-time cell analysis is an important functional indicator of cell viability, and cell index reflects changes in cell number and cell adhesion status. As shown in Figure 3C, the cell index of the blank group continues increasing. On the contrary, Ag@Se NPs and Ag@Se@RGD NPs caused the decrease of cell index. That is, the cell adhesion or viable cells number was significantly inhibited. Live/dead cell staining (Figure 4A) and MTT detection (Figure 4B) had similar results. NPs (0–60 μg/ml) caused the death of U251 cells in a dose-dependent manner. Ag@Se@RGD NPs at 20 μg/ml, 40 μg/ml and 60 μg/ml caused U251 cell death rates of 55.3, 60.8 and 64.5%, respectively. As the dosage increasing, the cell death rate did not increase significantly. Therefore, 20 μg/ml in subsequent experiments was used to verify the anti-tumor activity of Ag@Se@RGD NPs. Flow cytometry was used to explore the cell apoptosis. As shown in Figures 4C,D, Ag@Se@RGD NPs induced obvious apoptosis of U251 cells, and the number of apoptotic cells increased significantly with dose-dependent manner, which was mainly manifested by the increase of early apoptotic cells. Taken together, Ag@Se@RGD NPs inhibited glioma cells growth by induction of apoptosis.

### Ag@Se@RGD NPs Caused ROS Accumulation of U251 Cells

**FIGURE 5:**
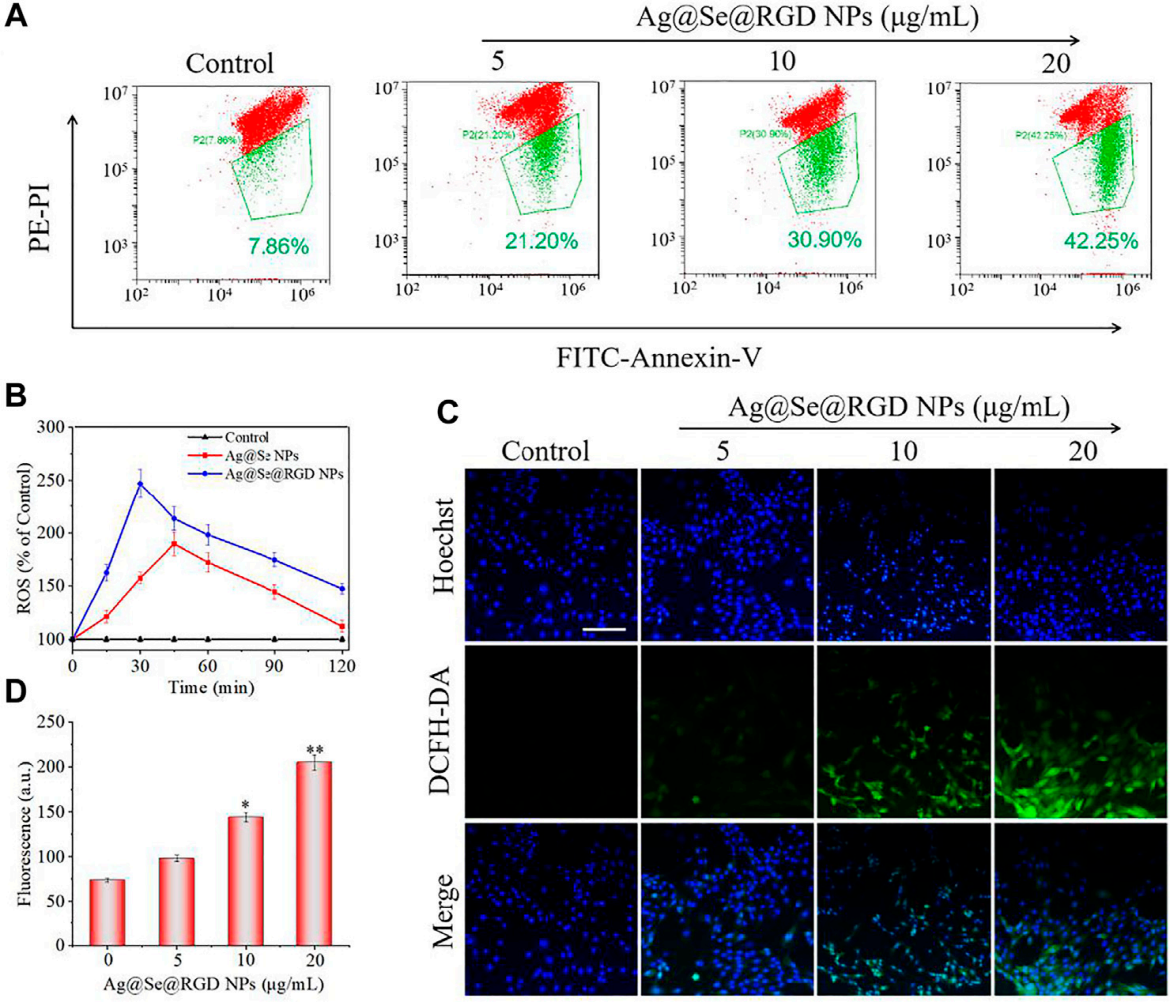
Ag@Se@RGD NPs caused ROS accumulation of U251 cells: **(A)** Glioma cells treated with Ag@Se@RGD NPs at 5, 10, and 20 μg/ml were measured for mitochondrial membrane potential by flow cytometry. **(B)** Quantitative analysis of ROS in glioma cells treated with Ag@Se NPs or Ag@Se@RGD NPs for 0–120 min. Fluorescence microscope imaging **(C)** and quantitative analysis of fluorescence intensity **(D)** of ROS when glioma cells were treated with Ag@Se@RGD NPs for 30 min. Scale bar: 50 μm.

The decrease of mitochondrial membrane potential was an important landmark event of early cell apoptosis (Carneiro and El-Deiry, 2020; Su et al., 2015). The result in Figure 5A showed that Ag@Se@RGD NPs treatment caused the significant loss of mitochondrial membrane potential with a dose-dependent manner. In the cell model, the DCFH-DA probe was used to check the production of intracellular ROS. As shown in Figure 5B, Ag@Se NPs treatment markedly increased the ROS level to 189% within 45 min, and the Ag@Se@RGD NPs increased the ROS content to 245% within 30 min, indicating that RGD modification promoted the absorption of NPs and resulted in the faster ROS production. As shown in Figures 5C,D, the ROS generation reflected by green fluorescence further confirmed Ag@Se@RGD NPs-induced ROS overproduction. Excessive production of ROS can cause damage to DNA and other signal factors (Aggarwal et al., 2019; Weinberg et al., 2019).

### Ag@Se@RGD NPs Triggered ROS-dependent MAPKs Activation

**FIGURE 6:**
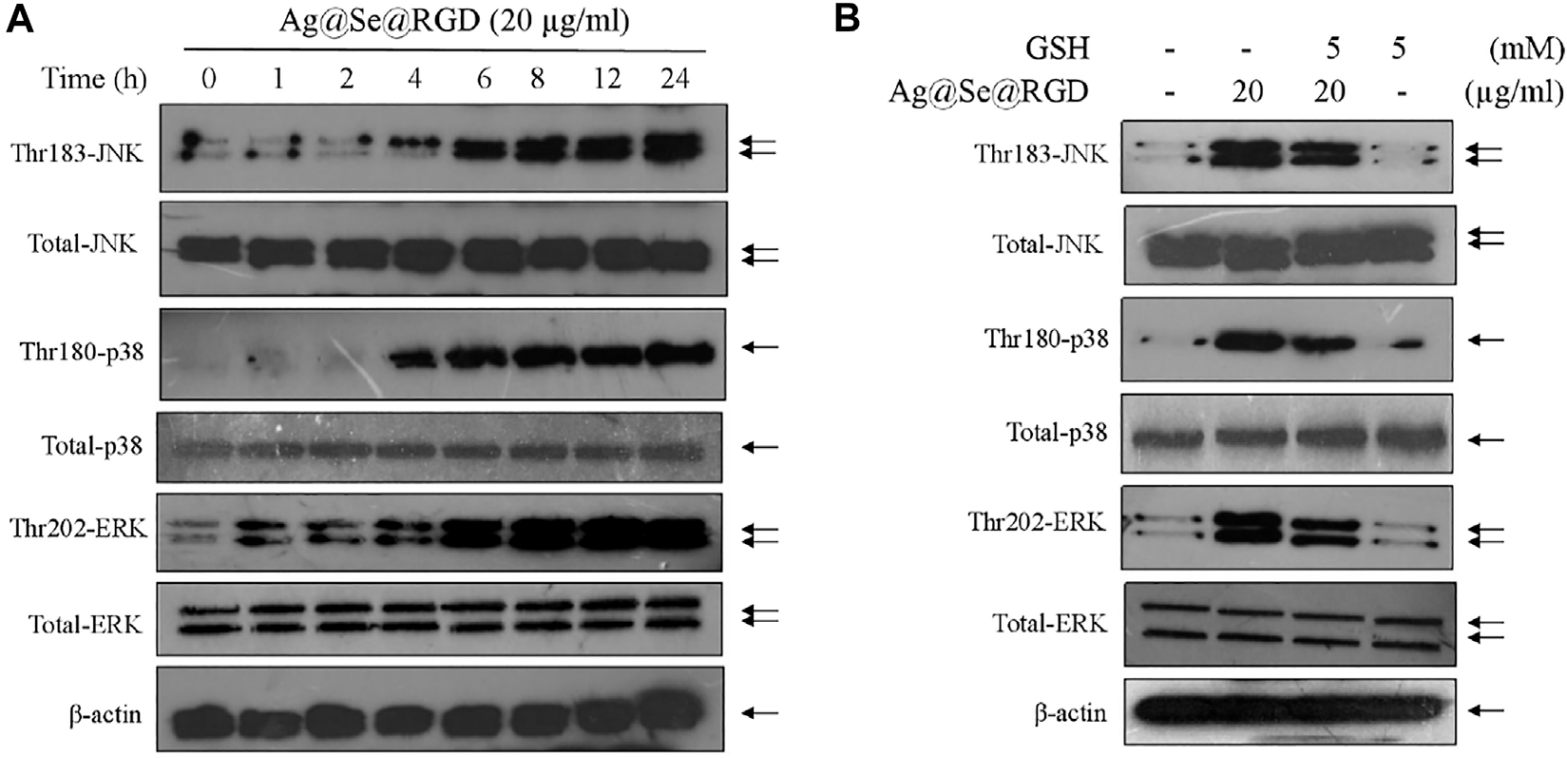
Ag@Se@RGD NPs triggered ROS-dependent MAPKs activation: **(A)** Ag@Se@RGD NPs triggered MAPKs activation. U251 cells were treated with 20 μg/ml Ag@Se@RGD NPs for 1–24 h. Protein expression was examined by western blotting method. **(B)** ROS inhibition attenuated Ag@Se@RGD NPs-induced MAPKs activation. U251 cells were pre-treated with 5 mM GSH, and co-treated with 20 μg/ml Ag@Se@RGD NPs for 24 h. Protein expression was examined by western blotting method.

MAPKs pathway can regulate cell growth, cell proliferation and cell division, which plays key role in drugs-induced apoptosis in human cancers (Sun et al., 2020; Sun et al., 2021). Herein, the three main components of MAPKs pathway, JNK, ERK and p38, were all examined to explore the underlying anticancer mechanism induced by Ag@Se@RGD NPs in U251 cells. As shown in Figure 6A, the time-course results revealed that Ag@Se@RGD NPs significantly increased the phosphorylation level of JNK (Thr183), ERK (Thr202) and p38 (Thr180) with a time-dependent manner, indicating that Ag@Se@RGD NPs treatment *in vitro* activated MAPKs pathway. The total JNK, ERK and p38 expression showed no significant changes. To elucidate the signal crosstalk between MAPKs pathway and ROS signal, ROS scavenge (glutathione, GSH) was employed. U251 cells were pre-treated with 5 mM GSH, and co-treated with 20 μg/ml Ag@Se@RGD NPs for 24 h, and the results showed that ROS inhibition by GSH effectively attenuated the phosphorylation level of JNK (Thr183), ERK (Thr202) and p38 (Thr180) in Ag@Se@RGD NPs-treated U251 cells, suggesting that ROS inhibition attenuated Ag@Se@RGD NPs-induced MAPKs activation. Taken together, these results suggested that Ag@Se@RGD NPs *in vitro* inhibited human glioma cells growth by triggering ROS-dependent MAPKs activation.

### Ag@Se@RGD NPs Effectively Crossed the BBB *in vitro*

**FIGURE 7:**
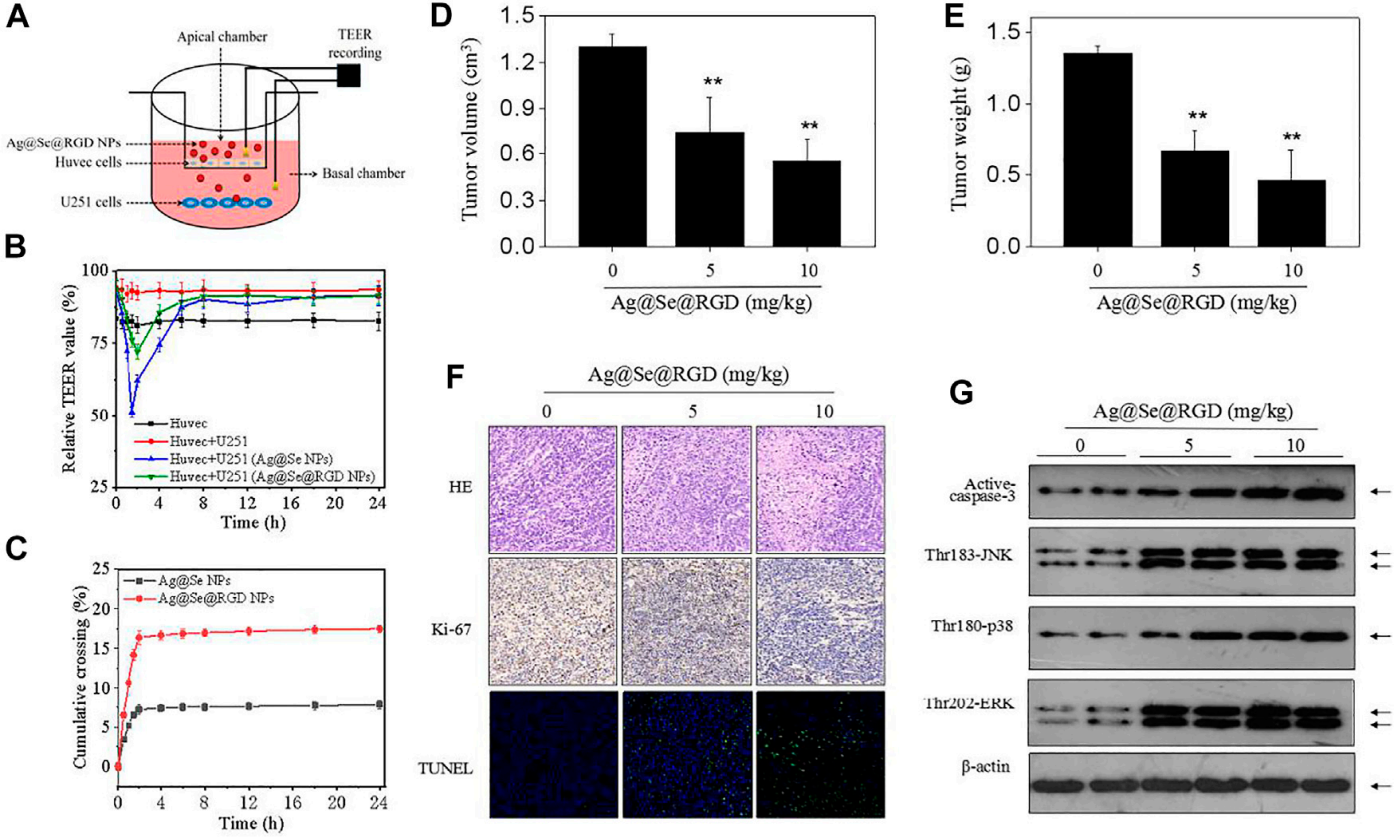
Ag@Se@RGD NPs inhibited tumors growth *in vivo*: **(A)** Blood brain barrier (BBB) model *in vitro*. **(B)** TEER value. The transendothelial cell electrical resistance (TEER) value measured immediately after addition of Ag@Se NPs or Ag@Se@RGD NPs (0–24 h). **(C)** Evaluating BBB crossing dynamics of Ag@Se NPs or Ag@Se@RGD NPs (0–24 h). **(D)** Tumor volume. **(E)** Tumor weight. Nude mice bearing U251 xenografts were administrated with Ag@Se@RGD NPs (5 and 10 mg/kg) every other day for 2 weeks. **(F)** Immunostaining of tumors. **(G)** MAPKs activation *in vivo*. MAPKs expression in tumor tissue was examined western blotting method.

To evaluate the possibility of Ag@Se@RGD NPs crossing the BBB, we quantitatively measured the BBB permeability using a *in vitro* BBB model (Figure 7A). The decrease of TEER value was related to the increase of the permeability of the cell layer barrier. As shown in Figure 7B, after 1.5–2 h treatment with Ag@Se@RGD, the TEER value was significantly reduced. The TEER value recovered about 6 h after the nanoparticles were treated, indicating that the change in the permeability of the BBB was a transient effect. Ag@Se@RGD NPs showed more higher BBB permeability rate, which was 2.24 times than that of Ag@Se NPs (Figure 7C). The results indicated that Ag@Se@RGD NPs had the potential to effectively cross the BBB.

### Ag@Se@RGD NPs Inhibited Tumors Growth *in vivo*

To further evaluate the anticancer potential of Ag@Se@RGD NPs, nude mice bearing U251 tumor xenografts were employed to explore the *in vivo* anticancer efficiency against human glioma. After 2-weeks administration, Ag@Se@RGD NPs (5 and 10 mg/kg) both significantly inhibited glioma growth *in vivo*, as convinced by the decreased tumor volume (Figure 7D) and tumor weight (Figure 7E). The *in vivo* anticancer mechanism induced by Ag@Se@RGD NPs was also investigated. The H&E and Ki-67 staining results showed that Ag@Se@RGD NPs *in vivo* significantly inhibited glioma nuclear heterogeneity and cell proliferation (Figure 7F). Ag@Se@RGD NPs administration *in vivo* also induced glioma cells apoptosis, as demonstrated by the increased TUNEL-positive cells (Figure 7F). Up-regulation of activ-caspase-3 expression further confirmed Ag@Se@RGD NPs-induced apoptosis *in vivo* (Figure 7G). Moreover, Ag@Se@RGD NPs *in vivo* markedly activated MAPKs pathway, as convinced by the increased phosphorylation level of JNK (Thr183), ERK (Thr202) and p38 (Thr180). Taken together, Ag@Se@RGD NPs inhibited glioma tumors growth *in vivo* by induction of apoptosis through regulation ROS-edpendent MAPKs activation. However, evaluation of anticancer effect in human glioma must consider the blood brain barrier (BBB). Hence, tumor-bearing nude mice by U251 cells subcutaneous injection conducted in the present study was not enough, and anticancer effect in glioma *in situ* should be further explored in future.

### *In vivo* Toxicity Evaluation of Ag@Se@RGD NPs

**FIGURE 8:**
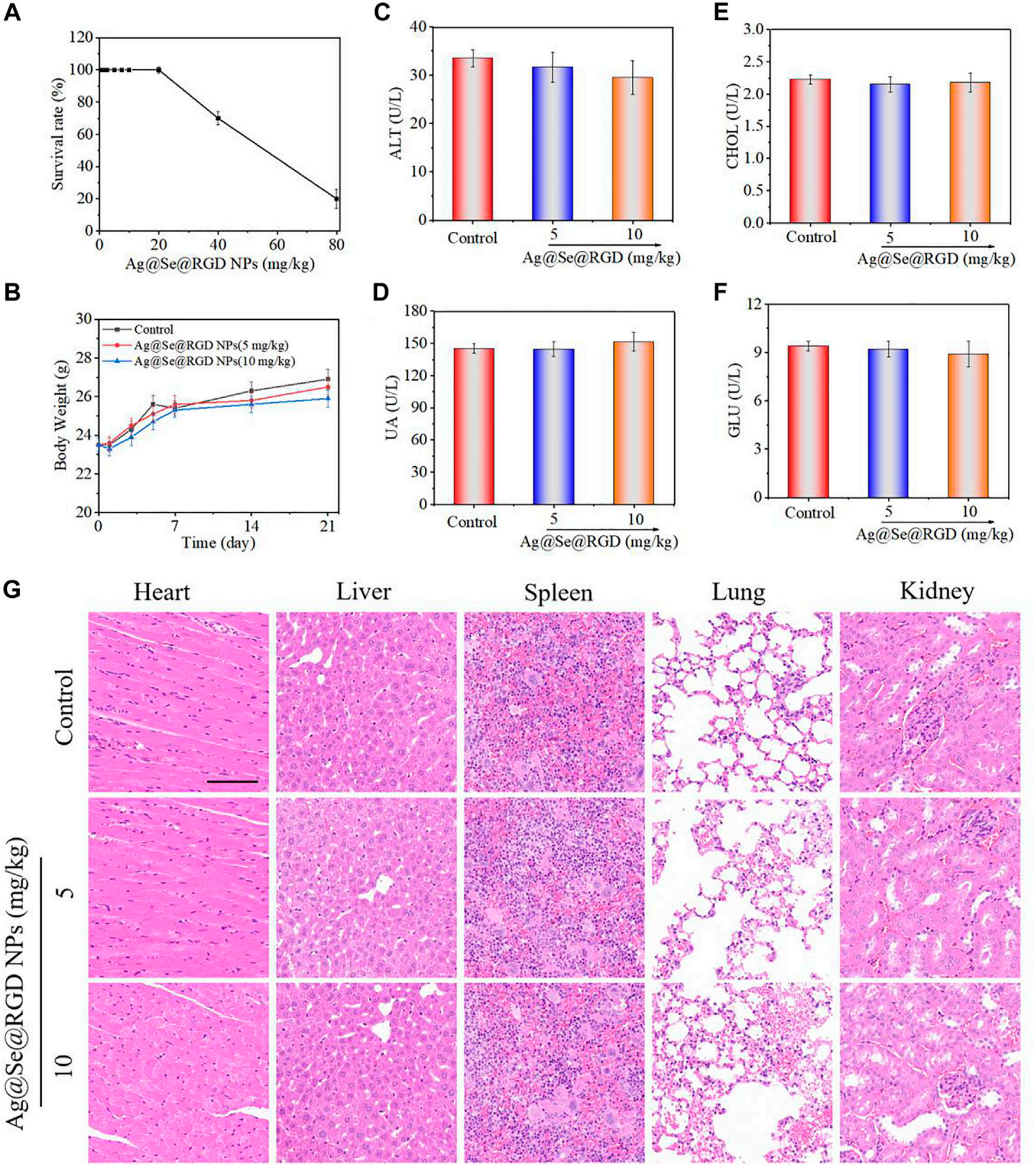
Safety evaluation of Ag@Se@RGD NPs. **(A)** Survival rate of mice. **(B)** Changes of mice body weight. Blood biochemical indicators include: liver function marker ALT **(C)**, renal function marker UA **(D)**, CHOL **(E)** and GLU **(F)**. **(G)** H&E staining for main organs. Scale bar: 100 μm.

As shown in Figures 8A,B, continuous treatment with 5 mg/kg or 10 mg/kg of NPs for 21 days has no significant effect on the body weight of the mice and the survival rate is 100%. In order to study the potential toxicity of Ag@Se@RGD NPs, the main organs and blood samples of mice were collected for H&E staining and blood biochemical testing. The levels of glutalanine aminotransferase (ALT), uric acid (UA), blood sugar (GLU) and cholesterol (CHOL) in mice treated with Au@Se@RGD NPs at 5 or 10 mg/kg were comparable to those of normal mice resemblance (Figures 8C–F). The H&E staining results (Figure 8G) of major organs also showed no obvious inflammation or damage, which proved the safety of Au@Se@RGD NPs.

## Conclusion

Ag@Se@RGD NPs were designed as cancer-targeted nano-drugs to achieve high-efficiency and synergistic tumor chemotherapy. NPs are a three-layer core-shell structure with a particle size of less than 100 nm, and exhibited tumor-targeted anti-tumor activity. Mechanism investigation revealed that Ag@Se@RGD NPs induced glioma cells ROS production, decreased mitochondrial membrane potential, and caused MAPKs activation, and ultimately resulted in tumor cell apoptosis. Our findings validated the rational design that Ag-Se NPs conjugated with RGD peptides was a promising strategy to combat human glioma by induction of apoptosis through triggering mitochondrial dysfunction and ROS-dependent MAPKs activation.

## Data Availability

The original contributions presented in the study are included in the article/Supplementary Material, further inquiries can be directed to the corresponding authors.
